# Carbon Nanotube Paper-Based Electroanalytical Devices

**DOI:** 10.3390/mi7040072

**Published:** 2016-04-20

**Authors:** Youngmi Koo, Vesselin N. Shanov, Yeoheung Yun

**Affiliations:** 1 FIT BEST Laboratory, Department of Chemical, Biological, and Bio Engineering, North Carolina A&T State University, Greensboro, NC 27411, USA; ykoo@ncat.edu; 2 NSF-Engineering Research Center, North Carolina A&T State University, Greensboro, NC 27411, USA; 3 Department of Chemical and Materials Engineering, University of Cincinnati, Cincinnati, OH 45221, USA; vesselin.shanov@uc.edu

**Keywords:** paper-based analytical device, carbon nanotubes (CNTs), HA-CNT sheets, nanostructure materials, origami paper device

## Abstract

Here, we report on carbon nanotube paper-based electroanalytical devices. A highly aligned-carbon nanotube (HA-CNT) array, grown using chemical vapor deposition (CVD), was processed to form bi-layered paper with an integrated cellulose-based Origami-chip as the electroanalytical device. We used an inverse-ordered fabrication method from a thick carbon nanotube (CNT) sheet to a thin CNT sheet. A 200-layered HA-CNT sheet and a 100-layered HA-CNT sheet are explored as a working electrode. The device was fabricated using the following methods: (1) cellulose-based paper was patterned using a wax printer, (2) electrical connection was made using a silver ink-based circuit printer, and (3) three electrodes were stacked on a 2D Origami cell. Electrochemical behavior was evaluated using electrochemical impedance spectroscopy (EIS) and cyclic voltammetry (CV). We believe that this platform could attract a great deal of interest for use in various chemical and biomedical applications.

## 1. Introduction

Paper-based analytical devices (PADs) have emerged as simple, yet powerful, platforms for performing low-cost, disposable, easy-to-use, and rapid analytical tests. They have been studied using various methods, including colorimetry [1,2,3,4], electrochemistry [5,6,7,8,9], chemiluminescence [10], electrochemiluminescence [11], and fluorescence [4,12], to detect analytes on paper-based platforms. Electrochemical detection has been explored in a paper-based platform due to its high accuracy and sensitivity [7,13,14,15,16]. PADs with integrated electrochemical detection are broadly explored. One technique involves directly printing electrodes onto paper. The paper is then placed onto a screen-printed electrode where various conductive inks such as Prussian blue, silver, and carbon are used [17,18,19]. These electrode materials combined with paper can play an important role on lowering detection limits and increasing sensitivity.

Carbon nanotubes (CNTs) are electrochemically inert materials, similar to other carbon-based materials used in electrochemistry, such as glassy carbon, graphite, and diamond. They also have good properties which include high surface area, high electrocatalytic effect, and fast electron transfer rate due to their unique sp^2^ electronic structure [20,21,22,23,24,25]. CNTs have become one of the most popular electrodes in the biosensor field [26,27,28,29]. Several studies have reported thorough proper control of their chemical and physical properties, as well as functionalization and immobilization, and multiple applications of this technology are possible. Some of these applications include use in glucose sensors [30]; biosensors for neurotransmitters/neurochemicals [31]; protein sensors for IgG [32] and IL-6 [33]; as well as DNA/RNA biomolecule sensors for the Influenza virus [34], Hepatitis C [35], and Hepatitis B [36].

Here, we present a new carbon nanotube (CNT) paper-based electroanalytical device using a highly aligned-CNT (HA-CNT) sheet integrated with cellulose paper. A free-standing HA-CNT sheet is fabricated in the form of a paper sheet with 200 layers that are inversely-ordered, where 100 of the layers play a role in the working electrode. The electrochemical system is integrated with an Origami cellulose paper. Furthermore, this paper evaluates the electrochemical properties of PADs using electrochemical impedance spectroscopy (EIS) and cyclic voltammetry (CV).

## 2. Materials and Methods

### 2.1. Materials

Phosphate buffered saline (1X PBS, without calcium and magnesium, pH 7.4, Cellgro, Manassas, VA, USA) solution containing 37 mM NaCl, 2.7 mM KCl, 10 mM Na_2_HPO_4_, 1.8 mM KH_2_PO_4_ and potassium ferricyanide (PF, 99+%, for analysis, ACROS Organics^TM^, Morris, NJ, USA) was purchased from Fisher Scientific. All chemicals were used as received without further purification. Silver foil (Alfa Aesar, Haverhill, MA, USA, 0.10 mm thickness, 99.998% pure, annealed) and platinum foil (Alfa Aesar, 0.127 mm thickness, 99.99%, Premion^®^) were used for reference and counter electrodes. Chromatographic cellulose filter paper (Whatman grade 1, 0.18 mm thickness, WhatmanTM, Florence, SC, USA) was used for the paper-based Origami cell construction. Circuit paper (AgIC International Corp., Tokyo, Japan) was used for printing the silver conductive pathway.

### 2.2. Preparation for Device Fabrication

**CNT sheets.** The free standing 200-layered HA-CNT sheet was drawn from a multi-wall carbon nanotube (MWCNT) array of 0.5 mm in height, grown using chemical vapor deposition (CVD), using our previously-reported method [23].

**Figure 1:**
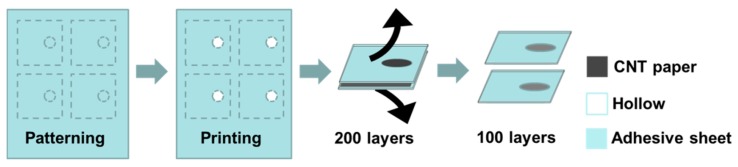
Inverse-ordered fabrication from 200-layered to 100-layered CNT sheet paper.

**Inversed-ordered thin layer CNT sheets.** In Figure 1, an electrode template was drawn using Silhouette Studio^®^ software (Silhouette America, Lehi, UT, USA) and adhesive sheets were cut using CAMEO print (Silhouette America). The inner size (open window) is 2 mm in diameter and the outer size (adhesive sheet) is 4 mm in width × 20 mm in length. The prepared 200-layered HA-CNT sheet was cut into the same size as the outer size of the adhesive sheet. Adhesive templates were sandwich-adhered to a 200-layered HA-CNT sheet and then were pulled into two 100-layered CNT sheet substrates [37].

**Figure 2:**
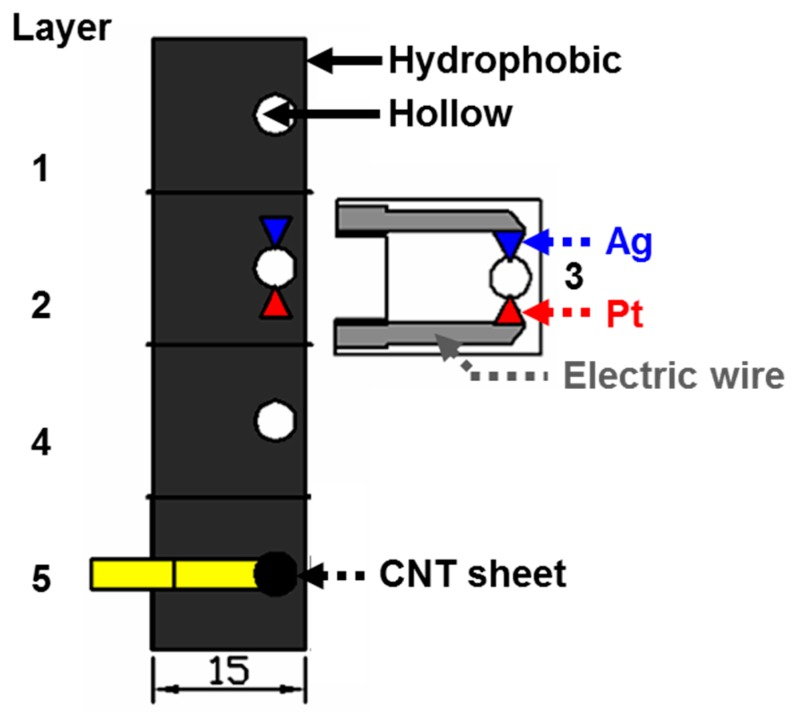
Basic design of the paper-based electroanalytical device.

**Paper-based electroanalytical device.** Three different printing technologies were used to fabricate a paper-based electroanalytical device. Designs were created using AutoCAD (Figure 2). An Origami paper-based chip (layers 1, 2, 4, 5) was printed onto filter paper using a wax printer (Xerox ColorQube 8570, Xerox, Norwalk, CT, USA). The Origami paper-based chip was then placed onto a hot plate (Super-Nuova, Thermo Scientific, Waltham, MA, USA) set to 123 °C for 5 min. The melted wax that was printed onto the Origami paper formed 3D-hydrophobic layers. Next, the hydrophilic region (white in color, as pictured in Figure 2) in the electrochemical chip was removed (hollow structure). Silver conductive patterns were printed onto layer 3 using a circuit printer (Brother, AgIC International Corp.). An as-prepared reference electrode, the blue triangle, and counter electrode, the red triangle, were place on both of the silver conductive patterns (electrical wires in Figure 2). All layers were affixed using double-sided adhesive tape, drawn using Silhouette Studio^®^ software, and were cut using the CAMEO Print & Cut. HA-CNT sheets as working electrodes were placed on the 5th layer of the Origami paper-based chip. A HA-CNT sheet was exposed as 2 mm in diameter through the holes in layers 1–4. Pressure was applied with a three-pound block that was placed a top of the assembled device, composed of a wax-printed Origami chip with a conductive pattern-printed layer, for 3 h.

### 2.3. Surface Morphology

The surface morphology and alignment of the multi-layered CNT sheet was characterized by field-emission scanning electron microscope (FE-SEM, Hitachi 8000, 5 kV, HITACHI, Atlanta, GA, USA).

### 2.4. Electrochemical Sensing Evaluation

The assembled paper-based electroanalytical device is composed of three electrodes embedded in a paper-based Origami cell. Electrochemical measurements of this device were performed where the HA-CNT sheet served as the working electrode, platinum served as the counter electrode, and silver served as the reference electrode. The three electrodes of this device were connected to a Reference 600^TM^ potentiostat (Gamry Instrument, Warminster, PA, USA). The electrochemical impedance spectroscopy (EIS) was obtained by conducting AC impedance measurements at a frequency range of 1.0 MHz to 0.2 Hz with 1X PBS and 5 mM K_3_[Fe(CN)_6_] in 1X PBS solution. The cyclic voltammetry (CV) was performed in 5 mM K_3_[Fe(CN)_6_] in 1X PBS solution. These measurements were carried out at room temperature. We dropped 20 μL of analyte to the reaction region in the paper-based chip.

## 3. Results and Discussion

**Figure 3:**
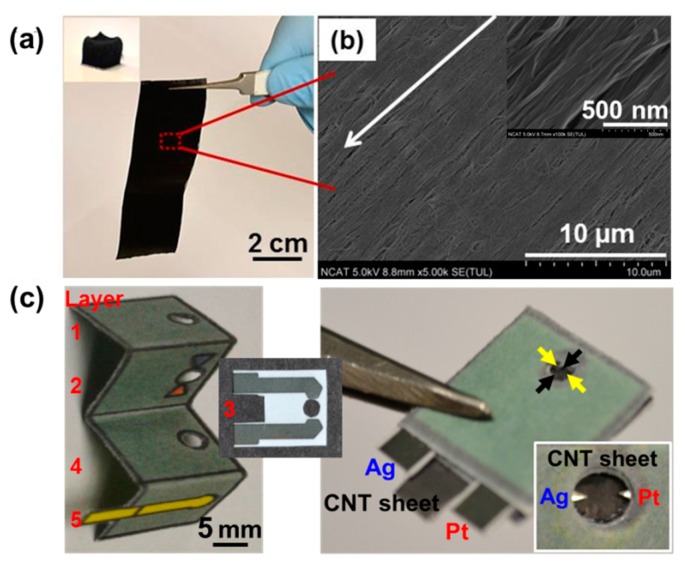
(**a**) Free standing HA-CNT sheet paper (insert: optical image of CNT array just before the CNT sheet is pulled out). (**b**) SEM image of the HA-CNT sheet paper (insert: enlarged SEM image). The white arrow demonstrates the alignment direction of the HA-CNT sheet paper and (**c**) optical images of the wax-printed Origami chip and conductive pattern-printed layer 3 and final assembled paper-based electroanalytical device (insert: the three electrodes).

Figure 3 shows the HA-CNT sheet as a working electrode and the final assembled paper-based electroanalytical device. In Figure 3a, the 200-layered CNT sheet was produced from a CNT array (insert of Figure 3a) showing a free standing and stable sheet. As shown in the SEM image in Figure 3b, the CNT sheet has a highly-aligned structure. The 100-layered HA-CNT sheet was fabricated by the aforementioned inverse-ordered method (Figure 1) with a pre-patterned, printed, adhesive sheet, and a thicker 200-layered CNT sheet. The CNT sheet is positioned as a working electrode inside a paper-based electrochemical cell (layer 5 of Figure 2). The wax-printed Origami chip and insert layer, including the reference and counter electrodes, were assembled with HA-CNT sheet paper (Figure 3c). The electrical resistance was <5.5 Ω (black arrow) and <12.5 Ω (yellow arrow) for the 200-layer HA-CNT sheet, <8.0 Ω (black arrow) and <12.2 Ω (yellow arrow) for the 100-layer sheet. Resistance was measured using an end-to-end method for the 2-mm-in-diameter circle. The parallel aligned direction’s resistance (white arrow shown in Figure 3b) was measured from one black arrow to the other black arrow, as pictured in Figure 3c. Resistance was also measured in the perpendicular direction, as indicated by the yellow arrows, also pictured in Figure 3c. Electrical resistance in the parallel direction is lower than the resistance from the perpendicular direction. The 200-layered HA-CNT sheet electrode showed high conductivity; however, conductivities of the perpendicular direction of alignment were observed to be similar, regardless of the thickness of the working electrodes. The final device’s dimensions were 15 mm in width × 15 mm in length × 1.2 mm in thickness. The insert of Figure 3c shows the electrochemical reaction zone of the paper-based electroanalytical device. For the 200-layered HA-CNT sheet, the working electrode is on the 5th layer, and the silver reference electrode and the platinum counter electrode is positioned on the 3rd layer. The total useable loaded sample volume is 20 μL.

**Figure 4:**
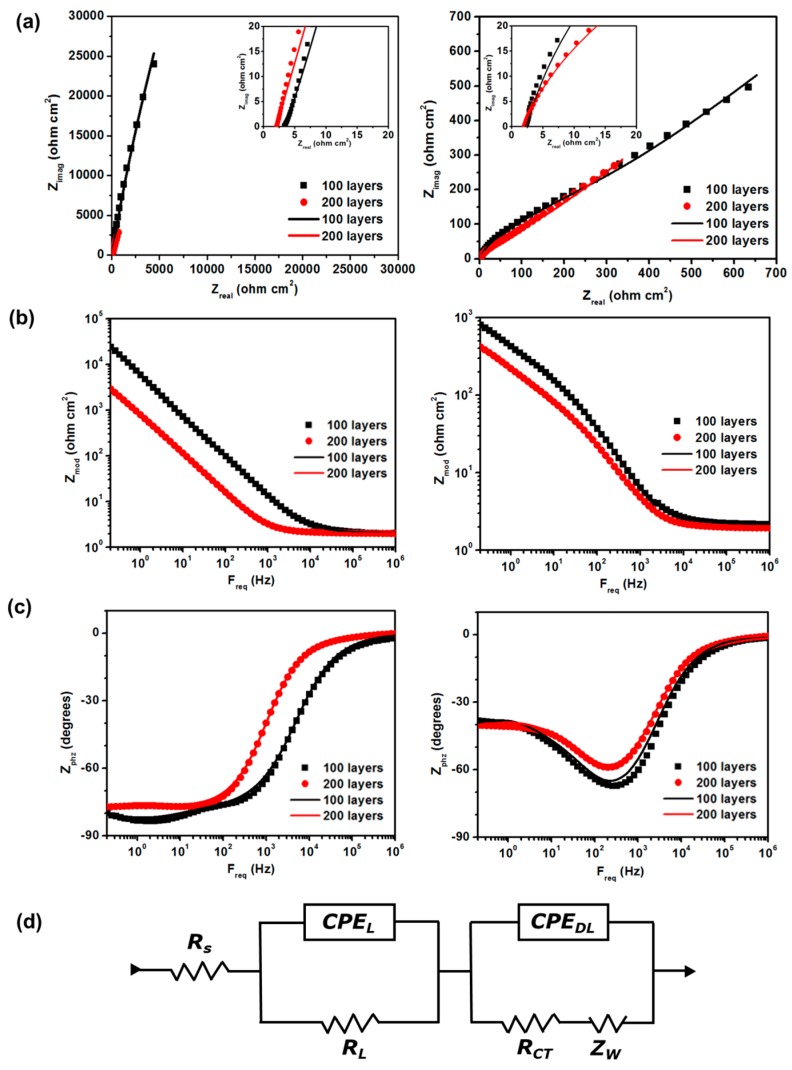
Impedance plots of paper-based electroanalytical device. (**a**) The Nyquist diagram, (**b**,**c**) Bode plots of the 200 and 100-layered HA-CNT sheet working electrodes between 1 MHz and 0.2 Hz frequency in 1X PBS (**left**) and 5 mM K_3_[Fe(CN)_6_] in 1X PBS solution (**right**) in an Origami paper-based chip. (**d**) Equivalent circuit for the impedance spectra of the paper-based electroanalytical device. The dots and lines noted in (a–c) represent experimental data and (d) a model of an equivalent circuit.

Figure 4 shows the impedance spectra (Nyquist and Bode plots) of the Origami chip integrated in the 200- and 100-layered HA-CNT sheets with electrolytes of 1X PBS (left) and 5 mM K_3_[Fe(CN)_6_] in 1X PBS solution (right). Figure 4d shows the equivalent circuit that consists of the following: R_s_, the electrolyte resistance, (the equivalent series resistance, ESR), CPE_L_ and R_L_, the capacitance and resistance of the 2 mm in diameter HA-CNT sheet working electrode, and CPE_DL_ and R_CT_, the double-layer capacitance and charge-transfer resistances, respectively [28]. Warburg impedance, Z_W_, is related to the ionic diffusion into the HA-CNT sheet. The fitted data for all circuit parameters are shown in Table 1. In the paper-based electrochemical chip, electrolyte resistances (R_s_) were similar, averaging 66 Ω (insert of Figure 4a). However, the resistance of the 200-layered HA-CNT sheet in the device was decreased in 5 mM K_3_[Fe(CN)_6_] as a redox probe in 1X PBS solution. The distinct semi-circle was not observed in the high-frequency region. Generally, it can be generated as the presence of an interface between the electrode, the current collector, and the electrical charge transfer in the electrode material. This shows that the HA-CNT sheet can act, either as a working electrode, or current collector inside the paper-based Origami electroanalytical chip for biosensors.

**Figure 5:**
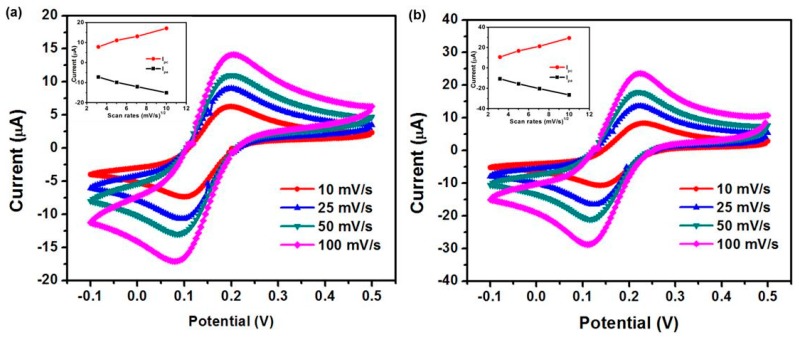
Cyclic voltamamograms on paper-based electroanalytical devices of the two working electrodes of different thicknesses in 5 mM K_3_[Fe(CN)_6_] as a redox probe in 1X PBS (pH 7.4) buffer at different scan rates. (**a**) 100-layered HA-CNT sheet, (**b**) 200-layered HA-CNT sheet. (Insert: shows plots of the redox peak currents *vs.* the square root of scan rate).

Figure 5 presents the cyclic voltammogram of paper-based electroanalytical devices that have two different layers of HA-CNT sheets as working electrodes. This analysis was performed to investigate the kinetics of the electrode reactions. The scan rate response of the electrode in the paper-based electroanalytical chip was carried out at different scan rates (10–100 mV/s) between +0.5 and −0.1 V in 5 mM K_3_[Fe(CN_)6_] in 1X PBS (pH 7.4) buffer. The results indicate oxidation and reduction peaks occur at 0.09 V and 0.2 V, respectively, with a linear equation of *I*_pa_ (μA) and *I*_pc_ (μA). Regardless of the number of layers (Figure 5a,b), the anodic potential shifts more towards the positive potential and the cathodic peak potential shifts in the reverse direction. The peak currents and the CV peak separation (∆*E*_p_) of Fe(CN)_6_^3−/4−^ were increased with the scan rates (Table 2). The results show that the peak currents were increased and the CV peak separation (∆*E*_p_) was decreased in the 200-layered HA-CNT device compared to that of the 100-layered device. The ratio of the reverse-to-forward peak currents, *I*_pa_/*I*_pc_, is almost equivalent for a redox couple. The diffusion coefficient for [Fe(CN)_6_]^3−^ was calculated according to the Randles-Sevcik equation [38]:

(1) $$\;i_{p}\;=\;0.4463nFAC{\left(\frac{nFvD}{RT}\right)}^{1/2}$$

where *F* is Faraday’s constant (96,485 *C*∙mol^−1^), *R* is the universal gas constant (8.314 J∙mol^−1^∙K^−1^), *i_p_* is the peak current in *A*, *n* is the number of electrons transferred, *A* is the electrode active surface area in cm^2^, *D* is the diffusion coefficient of the molecule in cm^2^∙s^−1^, *v* is the scan rate (V∙s^−1^), *C* is the concentration of the probe molecule, and *T* is temperature (K). The peak current was plotted against the square root of the scan rate (Figure 5 inserts). The slope of the linear fit (a = *i_p_/v^1/2^*) was used to determine the diffusion coefficient. The surface area of the HA-CNT sheet exposed as a working electrode was calculated (0.0314 cm^2^) based on the electrode’s geometry (Figure 1). The calculated diffusion coefficient was 9.0 × 10^−7^ cm^2^∙s^−1^ at the 100-layered HA-CNT sheet and 3.6 × 10^−6^ cm^2^∙s^−1^ at the 200-layered HA-CNT sheet, respectively. Redox of devices integrated within the two different layered working electrodes exhibited a high diffusion rate towards electrochemical oxidation. Current density (*I*) and potential separation (*E*_ps_) of devices composed of the 100-layered HA-CNT sheet as a working electrode also had a high sensitivity as noted from the CV results. For example, Taurino *et al.* reported a potential separation (*E*_ps_) 332.16 ± 0.02~446.26 ± 0.03 mV at 100 mV/s scan rate in 0.01 PBS containing 5 mM (K_4_Fe(CN)_6_) concentration [39]. Therefore, this paper-based electroanalytical device provides high peak-to-peak current (*I*_pa_ and *I*_pc_) and small potential separation (*E*_ps_), as seen in Table 2, which demonstrated a high sensitivity and lower detection limit. Furthermore, this HA-CNT paper-electrode with integrated cellulose paper can be functionalized directly with moieties such as carboxylic groups and biomolecules to its defect sites through covalent bonding [40,41]. It can also be modified with certain enzymes, antibodies and nucleotides [42] for point-of-care diagnostics.

## 4. Conclusions

We introduced the paper-based electroanalytical device consisting of cellulose-based paper, electrical connection patterned paper, and CNT sheet paper. The two different electrodes of 200- and 100-layered HA-CNT sheets were used as working electrodes in the Origami paper-based chip for the electroanalytical sensor. The 200-layered HA-CNT working electrode had electrochemically high conductivity, providing high sensitivity compared to that of the 100-layered HA-CNT. EIS and CV analysis was used with a simulated body fluid (1X PBS) solution as an electrolyte. However, the device consisting of the 100-layered HA-CNT sheet also showed a high sensitivity, based on CV analysis. Additionally, a paper-based Origami chip can be printed and directly applied for electrochemical measurements with a small sample for analysis.

## Figures and Tables

**Table 1:** Electrochemical parameters of equivalent circuits obtained from best fit to impedance data for paper-based electroanalytical devices.

Electrolyte	Layers	R_s_ (Ω)	CPE_L_ (S·s^*n*^)	*n*_1_	R_L_ (Ω)	CPE_DL_ (S·s^*n*^)	*n*_2_	R_CT_ (Ω)	Z_W_ (S·s^1/2^)
1X PBS	100	67.6	1.7 × 10^−5^	6.7 × 10^−1^	3.1 × 10^3^	8.9 × 10^−7^	9.4 × 10^−1^	1.7 × 10^5^	1.4 × 10^−7^
	200	65.02	6.7 × 10^−5^	8.0 × 10^−1^	3.1 × 10^3^	8.1 × 10^−6^	8.8 × 10^−1^	3.7 × 10^3^	4.0 × 10^−7^
5 mM PF in 1X PBS	100	69.15	3.0 × 10^−5^	6.8 × 10^−1^	4.2 × 10^3^	1.7 × 10^−6^	1.0 × 10^0^	1.3 × 10^3^	3.9 × 10^−5^
	200	62.12	4.2 × 10^3^	8.1 × 10^−1^	9.9 × 10^2^	1.5 × 10^−5^	8.1 × 10^−1^	4.2 × 10^3^	6.6 × 10^−5^

**Table 2:** Electrochemical data for 100- and 200-layered paper-based electroanalytical device by cyclic voltammetry at different sweeping rates.

Device	*v* (mV/s)	*E*_ps_ (mV)	*I*_pa_ (μA)	*I*_pc_ (μA)	*I*_pa_/*I*_pc_
100-layered WE	10	96	7.19	7.85	0.92
	25	103	9.83	11.1	0.89
	50	108	12	13.1	0.92
	100	119	15	17.1	0.88
200-layered WE	10	95	10.7	10.7	1.00
	25	98	15.6	16.8	0.93
	50	102	20.4	21.3	0.96
	100	111	26.5	29.4	0.90
